# Metabolic Imaging in Electrochemotherapy: Insights from FDG-PET Analysis in Metastatic Melanoma—A Pilot Study

**DOI:** 10.3390/cancers17162641

**Published:** 2025-08-13

**Authors:** Sophie C. Siegmund, Maximilian Deußing, Rudolf A. Werner, Daniela Hartmann, Christian Kunte

**Affiliations:** 1 Department of Nuclear Medicine, LMU University Hospital, LMU Munich, 81377 Munich, Germany; 2 Bavarian Cancer Research Center (BZKF), Partner Site Munich, Munich, Germany; 3 Department of Dermatology and Allergy, LMU University Hospital, LMU Munich, 80337 Munich, Germany; 4 Department of Dermatology, Allergology and Laser Medicine, Munich Municipal Hospital, 80337 Munich, Germany; 5 The Russell H Morgan Department of Radiology and Radiological Sciences, Division of Nuclear Medicine, Johns Hopkins School of Medicine, Baltimore, MD 21287, USA; 6 Department of Dermatology and Dermatosurgery, Artemed Fachklinik Munich, 80336 Munich, Germany

**Keywords:** FDG PET, electrochemotherapy, malignant melanoma, SUV

## Abstract

**Background/Objectives**: Electrochemotherapy (ECT) has emerged as a promising locoregional treatment modality for patients with cutaneous and subcutaneous melanoma metastases. While systemic therapies have improved overall disease control, effective local tumor management remains crucial, particularly in oligometastatic or symptomatic disease. This pilot study investigates the role of metabolic imaging with [^18^F]FDG PET/CT to assess tumor metabolism in melanoma patients undergoing ECT, building on prior evidence that PET offers valuable functional information beyond anatomical changes detected by conventional imaging. **Methods**: This retrospective study included 11 patients with histologically confirmed melanoma and cutaneous or subcutaneous metastases treated with ECT. [^18^F]FDG PET/CT scans were performed either before ECT, after ECT, or both. Metabolic response was assessed by measuring the tracer uptake (SUV_max_) of the ten hottest lesions. Morphological changes were evaluated using CT. Local progression-free survival was determined. **Results**: A total of 66 lesions were analyzed. Patients with PET/CT only after ECT showed significantly higher SUV_max_ and lesion size compared to those imaged before treatment (mean SUV_max_: 9.9 ± 11.2 vs. 10.3 ± 5.5; *p* = 0.034). Progression-free survival differed significantly based on pre-ECT SUV_max_ values (χ^2^ = 3.90; *p* = 0.048). Among two patients with follow-up imaging, one showed new lesions on CT with only mild FDG uptake, while the other developed newly FDG-avid metastases after ECT. **Conclusions**: FDG PET/CT provides valuable information on tumor viability and treatment response in melanoma patients undergoing ECT, demonstrated by significant differences in metabolic activity between lesions imaged before and after treatment. The lack of longitudinal intra-individual imaging limits definitive conclusions about the direct metabolic effects of ECT.

## Simple Summary

This pilot study explores the role of [^18^F]FDG PET/CT in assessing metabolic response to electrochemotherapy (ECT) in melanoma patients with cutaneous or subcutaneous metastases. In a retrospective cohort of 11 patients, significant differences in SUV_max_ between pre- and post-ECT lesions were observed, and higher pre-ECT metabolic activity was associated with shorter progression-free survival. While limited by small sample size, heterogeneity, and lack of longitudinal imaging, this is the first study to suggest FDG PET/CT as a complementary tool for evaluating ECT efficacy. The findings are hypothesis-generating and provide a rationale for future prospective studies to establish standardized imaging protocols and clarify the clinical utility of FDG PET/CT melanoma patients undergoing ECT.

## 1. Introduction

Melanoma is an aggressive form of skin cancer with a high potential for metastasis. Although systemic therapies have revolutionized the treatment landscape for malignant melanoma [1,2], locoregional treatment options remain crucial [3,4,5]. This is especially relevant given that 2–18% of melanoma patients develop cutaneous or subcutaneous metastases during the course of their disease [6,7], and 42-60% of metastatic melanoma patients present with widespread skin involvement [6,8]. Thus, locoregional treatments are particularly important for patients with oligometastatic disease, non-responders to systemic therapy, or those suffering from symptomatic cutaneous metastases. 

Electrochemotherapy (ECT) has emerged as a promising local treatment modality for cutaneous and subcutaneous melanoma metastases [9]. ECT combines the application of electric pulses (electroporation) with cytotoxic agents such as bleomycin or cisplatin, enhancing drug uptake by tumor cells and improving local tumor control [9,10,11,12]. In a large cohort study by Kunte et al., a complete response rate of 58% was reported for treated lesions in melanoma patients undergoing ECT, highlighting its effectiveness in achieving locoregional tumor control [13]. Beyond tumor control, ECT has also been effective in alleviating symptoms such as pain and ulceration [14]. Furthermore, ECT induces immunogenic cell death, which may synergize systemic immunotherapy to boost anti-tumor immune responses [15].

[^18^F]2fluoro-2-deoxy-D-glucose positron emission tomography/computed tomography (^18^F-FDG PET/CT) is widely used in oncology for staging, treatment response assessment, and prognosis. In melanoma, FDG PET offers valuable insights into tumor metabolism by visualizing glucose uptake, thereby complementing conventional morphological imaging methods such as CT [16]. Whereas CT assesses tumor size and structural changes, FDG PET detects metabolic alterations that often precede morphological responses, enabling earlier and potentially more accurate evaluation of treatment efficacy as demonstrated for other tumor entities [17,18,19,20].

Current guidelines recommend FDG PET for interim response assessment during immunomodulatory treatment in melanoma; however, its role in the context of ECT remains undefined [16]. FDG PET may also have a crucial role in pretherapeutic staging by identifying metabolically active lesions that could be missed or underestimated by CT alone, including deep-seated or clinically occult metastases. This capability can improve patient selection and treatment planning for ECT [17,21]. However, due to partial volume effects inherent to PET imaging, a Breslow thickness of at least 5 mm is generally considered the threshold for FDG PET positivity at initial staging [21,22].

A study in breast cancer patients has previously investigated the potential utility of FDG PET in the context of ECT, with a view toward monitoring treatment response [23]. However, data remain limited, and there is a lack of standardized response criteria specific to ECT. A more comprehensive understanding of the metabolic dynamics associated with ECT could contribute to optimizing patient selection and improving therapeutic strategies for metastatic melanoma.

The present study aims to investigate the role of metabolic imaging in melanoma patients undergoing ECT, guiding clinical decision-making. A better characterization of metastases may facilitate a more personalized approach to integrating ECT into the multimodal management of metastatic melanoma.

## 2. Materials and Methods

### 2.1. Study Design and Patients

This retrospective observational study was conducted at a tertiary cancer center. We included all patients with histologically confirmed malignant melanoma presenting with cutaneous or subcutaneous metastases who were eligible for ECT and underwent FDG PET/CT either (1) prior to, (2) after, or (3) both before and after ECT in our department. 

The study was conducted in accordance with the tenets of the Declaration of Helsinki and approved by the Institutional Ethics Committee of the Ludwig-Maximilians-Universität Munich (IRB #24-0974). Patient data collected included general clinical characteristics and tumor-specific parameters.

### 2.2. Radiopharmaceutical and Imaging Protocol

FDG was purchased commercially. FDG was injected in patients fasting for at least 6 h (blood glucose measurements were conducted) at a mean dose of 245 ± 20 MBq. All PET/CT scans were performed at the Department of Nuclear Medicine, LMU Munich, using the following EARL certified scanners: a Biograph mCT Flow scanner, a Biograph 64 PET/CT (Siemens Healthineers, Erlangen, Germany), or a GE Discovery 690 PET/CT. Image acquisition started on average after 64 ± 11 min. Patients received furosemide and butylscopolamine unless medically contraindicated, in order to improve image quality and ensure radiation protection. Contrast-enhanced diagnostic CT scans were acquired in the portal venous phase using 1.5 mL per kg body weight iopromide (Ultravist-300, Bayer Healthcare, Leverkusen, Germany). CT slice thickness was set to 0.3 cm. Image reconstruction was performed identically for all scans using an iterative TrueX algorithm (3 iterations, 21 subsets, and a 3D Gaussian post-filter with a 4 mm full width at half maximum). 

### 2.3. Electrochemotherapy

Electrochemotherapy was performed according to the ESOPE Standard Operating Procedures [24]. In short, Bleomycin was administrated intravenously using 15.000 IU/m^2^. General anesthesia was preferred for multiple metastases, large metastases (>3 cm), and metastases adhering to the periosteum or situated in sensitive regions (e.g. face and scalp), and in accordance with patient preference. Depending on the clinician’s choice, one of the following electrodes was used: 1) type II electrodes: two parallel rows of needles with 4 mm between rows; 2) type III electrodes: a hexagonal array with 7.9 mm between the needles. Electric pulses (eight pulses of 100 μs duration) were delivered using a square wave electroporator (IGEA, Carpi, Italy). The applied voltage relative to distance between electrodes was 1.3 kV/cm for plate electrodes and 1.0 kV/cm for needle electrodes, i.e., for the type II needle electrode with a 4 mm gap between the needles, the applied voltage was 400 V. For type II electrodes, the pulses are applied with 1 Hz or 5 kHz, whereas for type III electrodes, pulses can only be applied with 5 kHz. After electrochemotherapy, the treated metastases were covered with standard dressings where necessary.

Coverage of deep and lateral margins was not pre-specified but was at the treating physician’s discretion, and the database offered a possibility for the physician to indicate whether these respective margins were completely covered/treated or not [24,25,26].

### 2.4. Image Analysis 

PET was analyzed using dedicated software (Hermes Hybrid Viewer, Affinity 1.1.4; Hermes Medical Solutions, Stockholm, Sweden). Patients were divided into two groups based on the timing of their PET/CT scan: one group underwent imaging prior to ECT (pre-ECT), and the other group received imaging only after ECT (post-ECT). For each patient, a single-lesion analysis was conducted by assessing the maximum standardized uptake value (SUV_max_) of up to ten of the most metabolically active (hottest) cutaneous or subcutaneous lesions. The size of the corresponding lesions was determined on the basis of CT measurements of the short axis diameter (SAD) and the long axis diameter (LAD). The SUV_max_, SAD, and LAD of the lesions in both groups were then compared to evaluate differences in metabolic and morphological characteristics. Local progression-free survival (PFS) was defined as the time interval until the emergence of new cutaneous or subcutaneous metastases. In patients with pre-ECT and post-ECT PET, PET and CT scans were analyzed in regard to the presence of new metastases.

### 2.5. Statistical Analysis

The analysis was conducted in a descriptive manner using Microsoft Excel (Excel 2019, Microsoft, Redmond, WA, USA). The results obtained are presented as the mean ± standard deviation (SD). A Shapiro–Wilk normality test was conducted to ascertain the appropriate statistical analysis. Differences between groups were analyzed by unpaired *t*-test. The calculation of differences in progression-free survival was conducted by employing the use of Kaplan–Meier curves and the Chi-squared test. A *p*-value of <0.05 was defined as statistically significant.

## 3. Results

### 3.1. Patient Characteristics

A total of 11 patients were included in the study (mean age 67 ± 10 years; five females; six males). Histological subtypes were available for 7/11 patients: 2/11 patients had superficial spreading melanoma, 4/11 had nodular melanoma, and 1/11 had acral lentiginous melanoma. In the remaining 4/11 patients, the histological subtype could not be retrieved from the medical records. Initial TNM staging and systemic pretreatment regimens are summarized in Table 1. Three out of eleven patients received concurrent systemic therapy (two-thirds ipilimumab; one-third interferone alpha) at the time of ECT. PET/CT imaging was performed prior to ECT in 7/11 (63.6 %; mean 55 ± 33 days before ECT) and after ECT in 4/11 patients (36.4%; mean 61 ± 37 days after ECT). Two out of the seven patients with baseline PET/CT also underwent follow-up imaging after ECT. 

### 3.2. Lesion Detection and Imaging Characteristics

A total of 66 lesions were included in the analysis. Of these, 52/66 (78.8%) lesions were assessed in patients imaged before ECT and 14/66 (21.2%) lesions in patients imaged after ECT. In one out of seven (14.3%) patients in the pre-ECT group and two out of four (50.0%) patients in the post-ECT group, no measurable lesions were detectable in either PET or CT.

Overall, the mean SUV_max_ of all lesions was 9.9 ± 11.2. The mean SAD was 1.0 ± 0.6 cm, and the mean LAD was 1.4 ± 0.8 cm.

In patients imaged before ECT, the mean SUV_max_ was 9.8 ± 12.3, the mean SAD was 0.9 ± 0.6 cm, and the mean LAD was 1.3 ± 0.8 cm.

In patients imaged after ECT, the mean SUV_max_ was 10.3 ± 5.5, the mean SAD was 1.1 ± 0.4 cm, and the mean LAD was 1.6 ± 0.6 cm.

**Figure 1:**
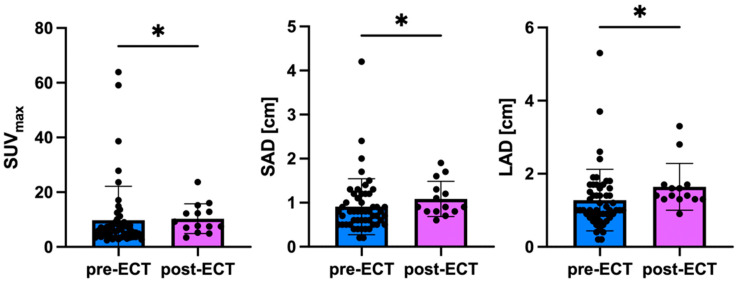
Imaging derived parameters pre-ECT and post-ECT. In total, 66 lesions were analyzed. Prior to ECT, 52/66 lesions were analyzed, and after ECT 14/66 lesions were. The mean SUV_max_ of all lesions in pre-ECT only PET/CT was 9.8 ± 12.3, the mean SAD 0.9 ± 0.6 cm, and the mean LAD 1.3 ± 0.8 cm. The mean SUV_max_ of all lesions in post-ECT only PET/CT was 10.3 ± 5.5, the mean SAD 1.1 ± 0.4 cm, and the mean LAD 1.6 ± 0.6 cm. Thus, the mean SUV_max_ (*p* = 0.034), the SAD (*p* = 0.039), and the LAD (*p* = 0.023) were significantly higher in post-ECT only PET/CT compared to pre-ECT only. ECT electrochemotherapy; LAD long axis diameter; SAD short axis diameter; * *p* < 0.05.

When comparing patients imaged only before ECT to those imaged only after ECT, the post-ECT group showed significantly higher mean SUV_max_ (*p* = 0.034), SAD (*p* = 0.039), and LAD (*p* = 0.023) (Figure 1, Table 2).

### 3.3. Impact of Concurrent Systemic Therapy

Among the patients imaged before ECT, one patient received ipilimumab concurrently. In this patient, the mean SUV_max_ was significantly higher compared to those without systemic therapy (*p* = 0.004), whereas there was no significant difference in SAD (*p* = 0.494) or LAD (*p* = 0.658).

### 3.4. Follow-Up Imaging

**Figure 2:**
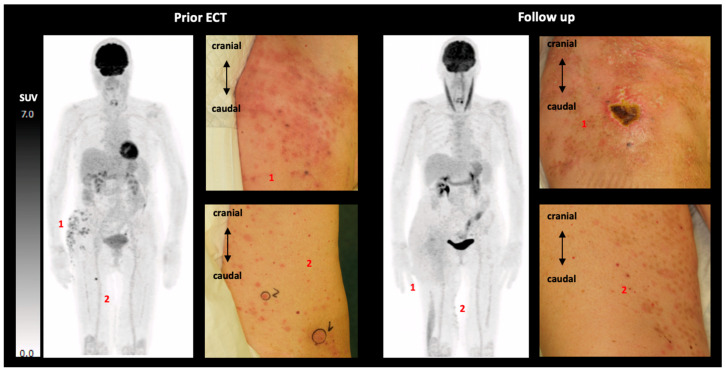
FDG PET imaging and clinical observations pre-ECT and at follow-up. The maximum intensity projection (MIP) prior to ECT revealed disseminated melanoma lesions at the right hip (1) and inner upper thigh (2) with a maximum SUV_max_ of 6.2, which was consistent with clinical observations (Pat. ID 4). However, metastases at the inner upper thigh showed only limited FDG uptake on PET. In the subsequent evaluation conducted approximately two months following the ECT, the presence of new lesions was identified on the PET scan, particularly in the inner upper thigh region (CT: maximum diameter 0.5 cm; PET: maximum SUV_max_ 2.0). However, progression was predominantly observed through clinical observation. ECT electrochemotherapy; MIP maximum intensity projection.

Two out of seven patients who underwent pre-ECT PET/CT also had follow-up imaging. In one case, CT imaging revealed new lesions with a maximum diameter of 0.5 cm with only faint FDG uptake (SUV_max_ 2.0; Figure 2). In the second case, new FDG-avid lesions were detected on PET.

### 3.5. Local Progression-Free Survival

**Figure 3:**
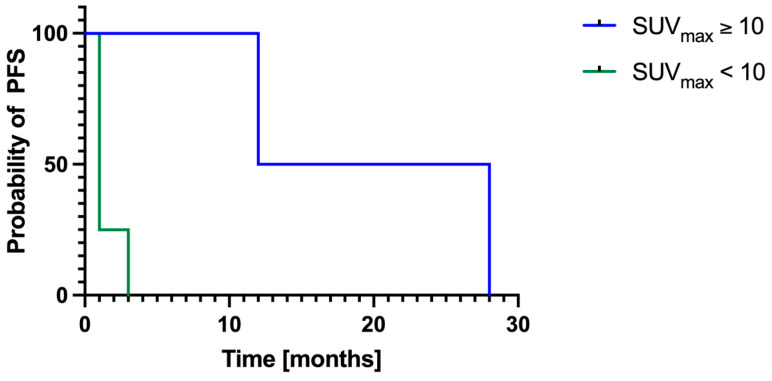
**Kaplan–Meier Curve.** Progression-free survival was available in nine patients. Six out of nine patients underwent pre-ECT imaging, and three out of nine patients underwent post-ECT imaging. Patients with a pre-ECT mean SUV_max_ ≥ 10 showed a significantly longer (χ^2^ = 3.90; *p* = 0.048) local progression-free survival (20 ± 11 months) compared to patients with a pre-ECT mean SUV_max_ < 10 (2 ± 1 months). ECT electrochemotherapy; PFS progression-free survival.

Follow-up data for local PFS were available in 9/11 patients. Of these, six out of nine had pre-ECT imaging and three out of nine had post-ECT imaging. The mean local PFS was 6 ± 9 months. Among patients with pre-ECT imaging, those with a mean SUV_max_ ≥ 10 had significantly longer (χ^2^ = 3.90; *p* = 0.048) local PFS (20 ± 11 months) compared to patients with mean SUV_max_ < 10 (2 ± 1 months; Figure 3).

## 4. Discussion

The data of this pilot study indicate that lesions assessed after ECT exhibited significantly higher metabolic activity (SUV_max_) and size parameters (SAD, LAD) compared to those assessed before ECT. Moreover, a higher pre-ECT SUV_max_ (≥10) was associated with significantly longer local progression-free survival, suggesting potential prognostic value. This study underscores the metabolic characteristics of melanoma lesions in relation to ECT, highlighting the necessity for further elucidation of the function of metabolic imaging during ECT. The observed disparities in metabolic activity between pre- and post-ECT lesions imply that FDG PET might offer valuable insights into tumor viability and treatment efficacy. Nevertheless, the lack of direct intra-individual pre- and post-ECT comparisons limits the ability to draw definitive conclusions about the metabolic effects of ECT on a per-patient basis.

### 4.1. Prognostic Value of Pre-ECT SUV_max_

Pre-treatment SUV_max_ may also serve as a surrogate marker of tumor aggressiveness or metabolic burden. Lesions with higher SUV_max_ could indicate a more immunologically active or treatment-responsive phenotype, potentially explaining the longer local progression-free survival observed in these patients following ECT. In contrast, lower SUV_max_ values may reflect more indolent or metabolically less active tumors. These findings highlight the potential of baseline metabolic activity to help stratify patients and guide treatment selection, although further validation in larger cohorts is required. 

### 4.2. Impact of Concurrent Systemic Therapy on FDG Uptake 

Systemic therapies, particularly immune checkpoint inhibitors, have been shown to influence FDG uptake in melanoma lesions [27,28]. In our cohort, the patient treated with ipilimumab concurrently with ECT showed a significantly higher SUV_max_ compared to those without systemic therapy, suggesting an interaction between immunotherapy and tumor metabolic activity. This observation aligns with previous reports indicating that immunotherapy-induced inflammation and immune cell infiltration can increase FDG uptake, complicating response assessment [29,30,31,32]. Anwar et al. reported that the appearance of new FDG avid lesions may be a more reliable indicator of treatment response than changes in SUV_max_, whereas Ito et al. suggested SUV_peak_ as a superior metric [33,34].

### 4.3. Interpretation of Post-ECT Findings

Patients who underwent post-ECT PET imaging exhibited significantly higher SUV_max_, SAD, and LAD values. This raises the question of whether these differences represent true disease progression, treatment-induced metabolic changes, or effects of ongoing systemic therapies. Coman et al.’s work on bone cell responses to ultrasound stimulation supports the concept that local therapies like ECT induce biologically meaningful changes, justifying the use of functional imaging such as FDG PET/CT to evaluate metabolic alterations after treatment [35]. Our findings reinforce the potential of metabolic imaging to capture early and dynamic treatment effects beyond anatomical changes and underscore the potential for future studies to integrate cellular-level assessments to further elucidate ECT mechanisms. 

### 4.4. Complementarity of FDG PET/CT and Conventional CT

FDG PET/CT and conventional CT offer complementary perspectives in evaluating tumor response. While CT assesses structural changes such as lesion size and morphology, FDG PET captures insights into metabolic activity, thus aiding in the detection of viable tumor tissue even when lesions appear stable or ambiguous on CT. In contrast, CT remains crucial for monitoring anatomical progression, particularly in lesions with low or absent FDG uptake. For example, one patient showed discernible lesions on CT with only faint FDG uptake (SUV_max_ 2.0). Our clinical observations showed that pigmented lesions subsequent to ECT revealed necrotic changes during biopsy, a finding that may provide a rationale for the reduced tracer uptake. Pretherapeutic imaging may be especially useful for identifying deep-seated lesions only visible on imaging, enabling the use of longer electrodes for optimized ECT application. Ultimately, the choice of imaging modality should be tailored to the clinical context—PET for early response assessment or unclear findings and CT for size-based monitoring.

### 4.5. Study Limitations

A key limitation is the small (n = 11) and highly heterogeneous patient cohort, which limits statistical power, particularly for PFS endpoints. Variability in imaging and treatment protocols, especially the timing of FDG PET/CT in relation to ECT, further complicates interpretation. The absence of longitudinal intra-individual imaging precluded robust conclusions about dynamic treatment effects over time. Moreover, histopathologic correlation of FDG positive lesions was lacking in most cases, restricting confirmation of imaging findings at the cellular level. The limited number of patients with sequential PET imaging after ECT hindered assessment of long-term metabolic changes and treatment efficacy. This lack of standardization reduces the reproducibility and interpretability of the results. Given the limited sample size and lack of adjustment for multiple comparisons, the reported *p*-values should be interpreted as exploratory rather than confirmatory, and future studies with larger cohorts are needed to validate these preliminary findings.

### 4.6. Clinical Heterogeneity and Real-World Implications

Direct comparison between pretreated and treatment-naïve patients are methodologically suboptimal but reflect real-world clinical practice. Patients frequently present with diverse prior therapies, making homogenous cohorts difficult to assemble. Our study offers a valuable foundation for future standardized research, especially since FDG PET/CT is not yet standard for ECT response assessment, making this dataset unique.

### 4.7. Comparison of PET and CT in Response Assessment

Few studies directly compare PET and CT findings in the same lesions before and after ECT. While PET provides functional insights, morphological changes detected by CT remain a crucial aspect of response assessment. In at least one case, PET failed to identify lesions visible on CT, underscoring the importance of multimodal imaging approaches. Another limitation is the exclusive use of SUV_max_ to evaluate metabolic response, which may not fully reflect the heterogeneity of tumor activity and treatment effects across lesions. Nevertheless, SUV_max_ is a widely accepted and commonly used PET parameter in different tumor entities [36,37].

### 4.8. Challenges in Long-Term Follow-Up

Limited long-term follow-up data—complicated by some patients receiving additional systemic therapies or multiple ECT cycles—makes interpreting metabolic changes over time difficult. Nonetheless, Kaplan–Meier analysis demonstrated significantly longer PFS in patients with a mean SUV_max_ ≥ 10 on pre-ECT PET/CT compared to those with a mean SUV_max_ < 10, suggesting that metabolic activity may influence response to ECT. Larger studies with standardized imaging protocols are needed to validate these findings.

### 4.9. Technical Considerations

When interpreting FDG PET findings, spatial resolution must be considered. Very small lesions may exhibit undetectable FDG uptake due to the partial volume effect, potentially leading to an underestimation of metabolically active disease [38]. This is particularly important when assessing response to ECT, as absence of FDG uptake does not necessarily indicate complete metabolic response but may instead reflect technical constraints of the imaging method. 

## 5. Conclusions

Acknowledging the limitations outlined above, the present findings suggest a potential complementary role for FDG PET/CT in assessing metabolic activity in melanoma lesions treated with ECT. However, given the current pilot nature of this study, the modest differences observed in SUV_max_, and the considerable interindividual variability, these results should be interpreted with caution. They are primarily hypothesis-generating and highlight the need for further validation in larger, prospective, multicenter studies with more homogeneous cohorts and standardized imaging protocols. Such research is essential to clarify the clinical utility of FDG PET/CT in this context, whether to optimize imaging strategies, avoid unnecessary procedures, or develop standardized imaging algorithms to guide treatment decisions.

## Figures and Tables

**Table 1:** Patient characteristics.

Pat. ID	Sex	Age	PET/CT	Systemic Treatment at ECT	Localization of ECT	Initial TNM Staging	Histological Subtype
1	F	66	prior ECT		gluteal	T1 N0 M0	n.a.
2	F	65	prior ECT		upper arm	T3 N1 M0	NM
3	F	59	after ECT		lower leg	TX N0 M0	n.a.
4	F	79	prior ECT		thigh, hip	T1 N0 M0	SSM
5	M	58	prior ECT		thigh	T3 N1 M0	n.a.
6	M	59	after ECT		parietal	T2a N2c M0	SSM
7	M	52	after ECT	INFalpha	thigh	T3b N3 M0	NM
8	F	82	prior ECT		lower leg	T4 N1 M1	ALM
9	M	77	prior ECT	Ipilimumab	arm	T4b N3 M1c	NM
10	M	65	after ECT	Ipilimumab	trunk, arm, abdomen, thigh	T3 N0 M1c	n.a.
11	M	76	prior ECT		trunk	T3b N1a M0	NM
**Mean**		**67**					
**SD**		**10**					

ALM acral lentiginous melanoma; ECT electrochemotherapy; F female; M male; n.a. not available; NM nodular melanoma; SD standard deviation; SSM superficial spreading melanoma.

**Table 2:** Overview of PET and CT lesional characteristics.

	SUV_max_	SAD	LAD
**Mean ± SD all lesions**	9.9 ± 11.2	1.0 ± 0.6	1.4 ± 0.8
**Mean ± SD lesions prior ECT**	9.8 ± 12.3	0.9 ± 0.6	1.3 ± 0.8
**Mean ± SD lesions after ECT**	10.3 ± 5.5	1.1 ± 0.4	1.6 ± 1.6

ECT electrochemotherapy; LAD long axis diameter; SAD short axis diameter; SD standard deviation.

## Data Availability

Data are available from the corresponding author upon reasonable request.

## Abbreviations

The following abbreviations are used in this manuscript:

ALM	acral lentiginous melanoma
CT	computed tomography
ECT	electrochemotherapy
F	female
FDG	[18F]2fluoro-2-deoxy-D-glucose
LAD	long axis diameter
M	male
n.a.	not available
NM	nodular melanoma
PET	positron emission tomography
PFS	progression-free survival
SAD	short axis diameter
SD	standard deviation
SSM	superficial spreading melanoma
SUV_max_	maximum standardized uptake value
